# Treatment decisions regarding caries and dental developmental defects in children - a questionnaire-based study among Norwegian dentists

**DOI:** 10.1186/s12903-019-0744-2

**Published:** 2019-05-10

**Authors:** M. M. Uhlen, H. Valen, L. S. Karlsen, A. B. Skaare, A. Bletsa, V. Ansteinsson, A. Mulic

**Affiliations:** 1 Oral Health Centre of Expertise in Eastern Norway (OHCE-E), Oslo, Norway; 2 Nordic Institute for Dental Materials (NIOM), Oslo, Norway; 3 Oral Health Centre of Expertise in Southern Norway (OHCE-S), Arendal, Norway; 4 Department of Paediatric Dentistry and Behavioural Science, Institute of Clinical Dentistry, University of Oslo, Oslo, Norway; 5 Oral Health Centre of Expertise in Western Norway Hordaland (OHCE-W Hordaland), Bergen, Norway

**Keywords:** Dental caries, Dental developmental defects, MIH, Hypomineralisation, Dental treatment, Restorative options, Treatment decisions

## Abstract

**Background:**

Current knowledge on treatment strategies and choice of restorative materials when treating deep caries or severe dental developmental defects (DDDs) in young individuals is scarce. Therefore, the aim was to investigate Norwegian dentists´ treatment decisions and reasons for treatment choice when treating deep caries in primary teeth and severe DDDs in permanent teeth in children.

**Methods:**

A pre-coded questionnaire was sent electronically to all dentists employed in the Public Dental Service (PDS) in Norway (*n* = 1294). The clinicians were asked about their background characteristics and how often they registered DDDs. Three clinical cases were presented to the dentists and asked to prioritize treatment options and reasons for their choice.

**Results:**

After three reminders, 45.8% of the dentists answered. Most clinicians were general practitioners (96.3%), females (77.9%), under 41 year-olds (59.4%), graduated in 2001 or later (61.1%), and representing all regions of Norway. The respondents registered molar incisor hypomineralisation (MIH), other DDDs and dental fluorosis (DF) frequently, 523 (91.1%), 257 (44.8%) and 158 (27.5%), respectively.

In case 1a with severe dental caries in a primary molar, the preferred treatment was resin-modified glass ionomer cement (RMGIC) (58.3%), followed by glass ionomer cement (GIC) (17.9%) and zinc oxide-eugenol (ZOE) (13.2%). Extraction, compomer or stainless steel crowns (SSC) were preferred by 0.9, 0.7 and 0.4%, respectively. In case 1b, which was identical to case 1a, but treated under general anaesthesia, the preferred treatment alternatives were RMGIC (37.1%), resin composite (RC) (17.6%) and GIC (17.2%). Extraction and SSC were chosen by 15.1 and 7.2%, respectively. In case 2, showing a severely hypomineralised and symptomatic first permanent molar, the dentists preferred RC (38.4%), followed by RMGIC (26.6%) and GIC (19.0%). Extraction and SSC were chosen by 8.7 and 5.4%, respectively. The treatment choices were not significantly affected by the dentists’ background characteristics. The reasons for dentists’ treatment decisions varied for each patient case; patient cooperation, prognosis of the tooth and own experience were the dominant reasons.

**Conclusions:**

A notable disparity in treatment choices was shown indicating that Norwegian dentists evaluate each case individually and base their decisions on what they consider best for the individual patient.

## Background

Dental caries is one of the most widespread chronic diseases regardless of age [1]. The distribution is skewed, and dental decay is mostly seen in societal groups with low socio-economic status and immigrant background in both the industrialised and non-industrialised world [2, 3]. Although dental caries is a largely preventable disease with well-known modifiable risk factors, caries management in young patients still represents a major task in everyday clinical practice [2]. Despite a documented reduction in the overall prevalence of dental caries [4], national data from Norway showed that 40% of 12-year-olds and 73% of 18-year-olds had caries or caries treatment experience in 2017 [5].

In addition to caries, developmental defects of the dental hard tissues are frequently observed in children and adolescents [6, 7].

The available treatment modalities of caries and dental developmental defects (DDDs) range from prevention to various forms of restorative treatment and extraction, and it may be difficult for clinicians to make the best treatment decision in both a short and long-term perspective [8]. Dental interventions to manage caries and DDDs can be challenging both for dentists to carry out successfully as well as for children to cope with [9]. The choice of intervention and the longevity of the restorations rely on several clinical variables, such as diagnosis and severity of the lesions, caries experience, type of tooth and surface and developmental status of the dentition. In addition, properties of dental materials, operator’s ability and patients’ characteristics like age, cooperation and oral hygiene must be taken into consideration [10–13]. For each case, the dentist must assess which treatment is most appropriate and thus, the treatment decision for a specific case is complex [14].

When operative therapy is required, there are several materials and techniques available [12, 15]. Restorations can be performed by various tooth-coloured materials (resin composite (RC), glass ionomer cement (GIC), resin-modified glass ionomer cement (RMGIC), polyacid-modified resin-based composites, or compomers (PAMRC). In addition, intermediate restorative materials based on zinc oxide-eugenol (ZOE, e.g. IRM®) may be used as a semi-permanent treatment. Re-establishing the original form of severely damaged teeth with a filling material can be difficult, particularly when multiple surfaces are affected. In some instances, restoration with preformed crowns (stainless steel crowns (SSC)) or extraction of severely affected molars with or without orthodontic intervention may be indicated [9, 13, 16].

In Norway, all children and adolescents are offered free comprehensive dental care from birth to 18 years of age in the Public Dental Service (PDS), and almost all (97,6%)of the children are enrolled in the services [17]. Therefore, the majority of dentists working in the PDS in Norway are general practitioners [17]. However, oral health care for adults is mostly provided by private practitioners [18].

As dental health professionals, in the PDS examine children and adolescents on a regular basis, and are in a unique position to make an early diagnosis of oral diseases as well as DDDs before extensive breakdown. The dentist working in the PDS are therefore responsible for the initial management of both dental caries and DDDs of children. However, current information on treatment strategies and choice of materials when treating children is scarce. Therefore, the aim of this questionnaire-based study was to investigate the dentists´ treatment decisions and reasons for their choices when treating deep caries in primary teeth and severe DDDs in permanent teeth in children.

## Methods

In May 2018, a pre-coded questionnaire was sent electronically to all dentists employed in the PDS in Norway using the software Questback [19]. Of 1407 dentists employed in the PDS in 2017 [5], email addresses from 1294 (91.97%) dentists were obtained from the Chief Dental Officers in the 18 Norwegian counties. The questionnaire software was configured to automatically send up to three reminders to participants who did not respond within a reasonable time (the questionnaire is available upon request to the corresponding author).

**Fig. 1:**
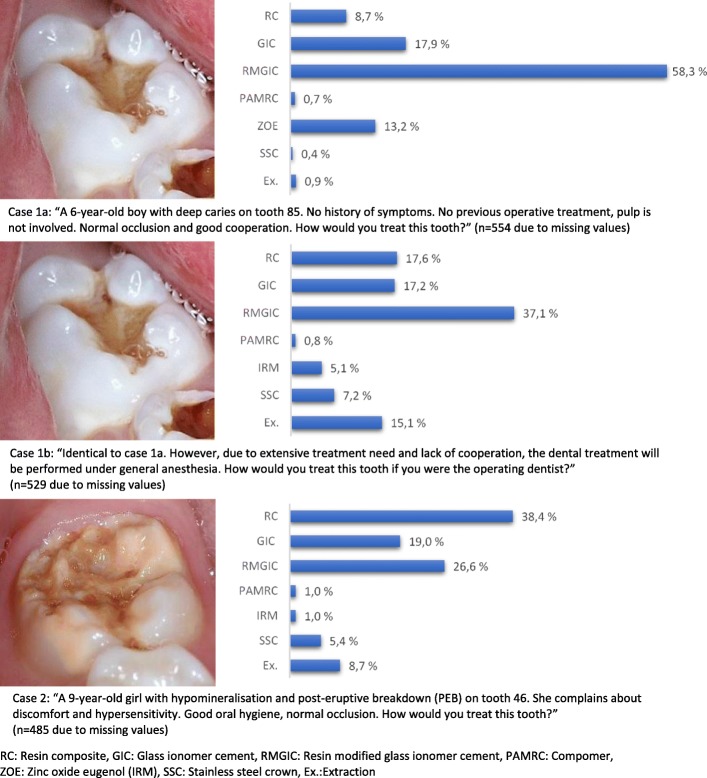
Case 1a, 1b and 2. Preferred choice of treatment among the respondents

The main items in the questionnaire were:

- Background characteristics of dentists: Gender, age, county of practice and full-time or part-time occupation in PDS, year and country of graduation, speciality, and to which extent the respondents worked clinically and were involved in the diagnosis and treatment of children and adolescents
- How often the respondents registered DDDs in their patients (aged ≤20 years old), with the given alternatives: molar incisor hypomineralisation (MIH), dental fluorosis (DF) or other DDDs.
- Three cases (1a, 1b and 2) illustrated by a clinical photograph and a brief patient history were presented for the dentists to range the different treatment alternatives and reason for choice of treatment in a prioritised order (Fig. 1). Case 1a and 1b illustrated the same patient: A 6-year-old child with asymptomatic, deep occlusal caries in a primary molar. In case 1b, the dental treatment was planned to be performed under general anaesthesia (GA) due to extensive treatment needs and lack of cooperation, but otherwise identical to case 1a. Case 2 showed a severely hypomineralised and symptomatic first permanent molar in a 9-year-old child. Statistical analysis

Data were processed and analysed using SPSS statistical program package (IBM SPSS 25.0, SPSS Inc., Chicago, IL, USA). Frequency distributions were used for descriptive statistics and Chi-squared test was used to test bivariate associations. A significance level of 5% was used throughout.

### Ethical considerations

Participation was voluntary, and no compensation was given to the respondents. Anonymity of the respondents was ensured by QuestBack. The study was approved by the Norwegian Social Science Data Services (ref.no. 57710).

## Results

Replies were received from 614 dentists after three reminders. Dentists neither clinically active nor working with paediatric patients (*n* = 40) were excluded. Thus, answers from 574 respondents were further processed in the final statistical analyses. A response rate of 45.8% was calculated according to the Standard Definitions of the American Association for Public Opinion Research [20].

Background characteristics of the dentists are presented in Table 1 with regard to gender, age, regional affiliation, year and country of graduation, main occupation and speciality. Data on gender and age for all PDS-employed dentists were extracted from Statistics Norway, Dental Health (SSB) [5]. The study sample was not statistically significantly different from the data extracted from SSB regarding gender (*p* = 0.2) and age (*p* = 0.3). Replies were obtained from dentists working in all regions of Norway. The majority of the responding dentists graduated in 2001 or later, and most of the respondents graduated from Nordic countries (Table 1). Almost all respondents had their main occupation (50% of working time or more) in the PDS. Most of the dentists worked as general practitioners, and only 21 (3.7%) had a postgraduate speciality (paediatric dentistry, prosthodontics, orthodontics, endodontics, oral radiology, oral surgery or periodontology) (Table 1).

**Table 1:** Background characteristics of dental personnel in the study (*n* = 574)

	n	%
Gender		
Female	447	77.9
Male	127	22.1
Age		
< 30	81	14.1
30–40	260	45.3
41–50	122	21.3
51–60	67	11.7
> 60	44	7.7
Region		
East	136	23.7
Oslo region	90	15.7
South	50	8.7
West	140	24.4
Middle	73	12.7
North	85	14.8
Year of graduation		
< 2001	189	32.9
≥ 2001	385	67.1
Country of graduation		
Nordic country	447	77.9
Other	127	22.1
Full-time or part-time occupation in Public dental service (PDS)		
≥ 50% PDS	568	99.0
Other	6	1.0
General practitioners	553	96.3
Specialist		
Paediatric dentistry	7	1.2
Prosthodontics	5	0.9
Orthodontics	4	0.7
Other	5	0.9

Almost all respondents, 523 (91.1%), reported to register MIH frequently (weekly/monthly), nearly half 257 (44.8%), registered other DDDs frequently, while one third, 158 (27.5%), registered DF frequently.

In case 1a, the preferred treatment option among more than half of the respondents was RMGIC, followed by GIC and ZOE (Fig. 1). Very few clinicians preferred extraction, compomer or SSC as their treatment of choice for this patient.

In case 1b, the treatment decisions were more evenly distributed among the respondents. As in case 1a, the most frequently preferred treatment alternative was filling with RMGIC. Extraction was a more common treatment alternative in case 1b compared to case 1a, chosen almost as frequently as restoration with RC and GIC. Although case 1a and 1b were illustrated by the same clinical photograph, a larger proportion of the dentists (7.2% vs 0.4%) preferred SSC when the treatment was planned to take place under GA.

In case 2, the most frequently chosen treatment was filling with RC, followed by RMGIC and GIC. Extraction of the tooth and SSC were the preferred choice by 8.7 and 5.4% of the respondents, respectively. In all three cases, only 1% of the respondents or less would choose compomer as their preferred treatment material.

The dentists were asked to range the reasons for their treatment decisions in each case. The eight alternatives were aesthetics, own experience, time available, the cooperation of the patient, materials available, number of affected molars, their perception of the prognosis of the tooth and the longevity of the material. However, in case 1b, cooperation was not an alternative because of treatment under GA.

In case 1a, patient cooperation, prognosis of the tooth and own experience were the dominant reported reasons, regardless of treatment choice (Table 2). In case 1b, prognosis of the tooth and own experience were the main reasons when deciding the treatment. Material longevity was the dominant reason for treatment choice only for the clinicians selecting SSC.

**Table 2:** Reasons for preferred treatment

Treatment option	Reasons for preferred treatment	n (%)	Total
Case 1a			
RMGIC (58.3%)	Patient cooperation	182 (56.7)	321
	Prognosis of the tooth	100 (31.2)	
	Experience	25 (7.8)	
GIC (17.9%)	Patient cooperation	52 (52.5)	99
	Prognosis of the tooth	34 (34.3)	
	Experience	7 (7.1)	
ZOE (13.2%)	Patient cooperation	38 (53.5)	71
	Prognosis of the tooth	25 (35.2)	
	Experience	8 (11.3)	
RC (8.7%)	Patient cooperation	24 (52.2)	46
	Prognosis of the tooth	17 (37.0)	
	Experience	3 (6.5)	
Extraction (0.9%)	Aesthetics	3 (60.0)	5
	Patient cooperation/prognosis of the tooth	2 (40.0)	
PAMRC (0.7%)	Prognosis of the tooth	4 (100.0)	4
SSC (0.4%)	Patient cooperation/number of affected molars	2 (100.0)	2
Case 1b			
RMGIC (37.1%)	Prognosis of the tooth	147 (78.6)	187
	Experience	22 (11.8)	
	Time available	7 (3.7)	
RC (17.6%)	Prognosis of the tooth	66 (72.5)	91
	Experience	14 (15.4)	
	Number of affected molars	7 (7.7)	
GIC (17.2%)	Prognosis of the tooth	59 (67.0)	88
	Experience	11 (12.5)	
	Number of affected molars	9 (10.2)	
Extraction (15.1%)	Prognosis of the tooth	62 (80.5)	77
	Experience	10 (13.0)	
	Number of affected molars	2 (2.6)	
SSC (7.2%)	Prognosis of the tooth	19 (51.4)	37
	Experience	8 (21.6)	
	Material longevity	5 (13.5)	
ZOE (5.1%)	Prognosis of the tooth	19 (70.4)	27
	Experience	5 (18.5)	
	Number of affected molars/materials available/aesthetics	3 (11.1)	
PAMRC (0.8%)	Prognosis of the tooth	2 (50.0)	4
	Materials available/experience	2 (50.0)	
Case 2			
RC (38.4%)	Patient cooperation	81 (44.0)	184
	Prognosis of the tooth	81 (44.0)	
	Experience	18 (10.0)	
RMGIC (26.6%)	Prognosis of the tooth	57 (44.9)	127
	Patient cooperation	49 (38.6)	
	Experience	12 (9.4)	
GIC (19.0%)	Prognosis of the tooth	38 (42.0)	91
	Patient cooperation	33 (36.3)	
	Experience	15 (16.5)	
Extraction (8.7%)	Prognosis of the tooth	29 (69.1)	42
	Experience	6 (14.3)	
	Patient cooperation	3 (7.1)	
SSC (5.4%)	Prognosis of the tooth	10 (38.5)	26
	Patient cooperation	8 (31.0)	
	Number of affected molars	5 (19.2)	
ZOE (1.0%)	Patient cooperation/experience	4 (80.0)	5
	Number of affected molars	1 (20.0)	
PAMRC (1.0%)	Prognosis of the tooth	2 (40.0)	5
	Number of affected molars/aesthetics/patient cooperation	3 (60.0)	

The three highest prioritised reasons for the most preferred choice of treatment in each clinical case (1a, 1b and 2). Different n due to missing values

Regarding case 2, patient cooperation, prognosis of the tooth and own experience were, as in case 1a, the main reasons for treatment choice. However, in contrast to case 1a, own experience and number of affected molars were reported as reasons for choosing extraction and SSC, respectively.

## Discussion

In the present study, almost all dentists reported to observe MIH frequently whereas nearly half registered other DDDs and one third recorded DF as often. As caries rates have declined in western countries during last decades, DDDs may be more apparent requiring more complex and long-term treatment options [14]. All dentists in this study were practicing in the PDS and treating children at a daily basis. Hence, they may see their patients when teeth are newly erupted, in contrast to general practitioners in private sector in Norway which mostly treat adult patients, who probably would record a lower number due to restored or extracted teeth.

In Norway, an estimated prevalence of dental fluorosis is reported to be 25% [21]. The main cause for mild DF is high consumption of fluoride toothpastes and fluoride supplements [22], while severe DF is generally seen in individuals growing up in areas or countries where the drinking water has high fluoride content [23]. Almost half of the respondents recorded DDDs frequently, which is not surprisingly since they may be manifestations of a local insult (trauma or idiopathic), of systemic origin (MIH) or inherited (AI). A recent study [6] reported the prevalence of any type of developmental defects of enamel (DDE) to be 33.2%, somewhat lower than other comparable studies.

Restorative caries treatment aims to replace missing dental tissue and restore tooth function, to protect the pulp-dentine complex by sealing the cavity and to aid plaque control [24]. Although a wide selection of restorative materials is available, they all have a limited lifespan. Although the properties of dental materials are well-known, information on treatment strategies and choice of materials when treating deep caries and severe DDDs in children and adolescents is still scarce.

The results of the present study showed a notable disparity between clinicians’ treatment decisions. More than half of the responding dentists preferred RMGIC and less than 1% chose extraction or SSC in case 1a. However, for case 1b, fewer dentists chose RMGIC and more preferred extraction. One may speculate that the higher proportion of reporting extraction for the patient under GA was for two reasons; partly to avoid repeated GA in a patient with extensive treatment need and partly to the challenge of extracting a severely damaged tooth in a young child with poor cooperation. In addition, more dentists also reported to prefer SSCs when treating the same tooth and patient under GA (7.2% in case 1a vs 0.4% in case 1b). This is in accordance with the indications for SSCs and is supported by other studies both recommending SSCs in children receiving dental treatment under GA [15, 25], as well as showing more frequent use of SSCs when treatment is performed under sedation or GA [26].

It is also worthy to mention that ZOE based materials are used for stepwise excavation and to postpone the final restoration, and is a frequent choice, especially in primary teeth. Therefore the alternative was given to the responders.

For case 2, the most frequently preferred treatment alternative was filling with RC, followed by RMGIC and GIC. RCs have increased survival and success rate compared with other restorative materials in MIH teeth [14, 27, 28]. Instead of restorative treatment, 8.7% of the dentists preferred extraction, which is a good alternative in severely damaged first permanent molars, in cases of frequently repeated treatments or when pulpal symptoms are hard to cure [16, 29]. However, any extraction of a first permanent molar should only be carried out with the possible orthodontic complications in mind [29]. A well-timed extraction could yield spontaneous space reduction and favourable development of the permanent dentition [14]. SSCs were reported as the preferred treatment option by 5.7% of the responding dentists although being a recommended treatment option to provide full coverage of defective molars [26]. Theoretical and practical education about SSCs is implemented in the curriculum for all dental students in Norway, but the clinical training during the education is generally low, possibly due to low caries prevalence. This could contribute to the fact that very few dentists in this study reported to prefer SSCs as their first treatment option for the three cases.

In the present study the clinicians based their treatment decisions mostly on patient cooperation, prognosis of the tooth and own experience. However, in addition, aesthetics and number of affected molars were reasons when extraction and SSC were preferred as treatments, respectively. Material longevity was a dominant reason only when SSCs was the preferred treatment choice. Relatively few clinicians used or were aware of SSC, and the reason for this should be explored further. In addition it is difficult to assume the reason why esthetics was chosen by dentists for the extraction and ZOE. However, it should be kept in mind that only 3 dentist chose esthetics when considering extraction. And only 3 dentists chose number of affected molars/materials available/aesthetics for ZOE.

It can be argued that the reported attitudes and routines do not necessarily reflect actual behaviour, and that the response rate could be higher. However, as most web surveys today usually have modest response rates, the response rate of 45.8% is considered acceptable [30]. In addition, most of the clinicians were general practitioners, females, under 41 years old, graduated in 2001 or later, and working in all regions of Norway. Gender and age distribution of our sample was representative of all PDS-employed dentists in Norway. However, the treatment choices were not significantly affected by their background characteristics.

The present study was questionnaire-based, and study participants self-selected to complete the survey, thus selection bias related to personal interests of clinicians may have occurred. Furthermore, recall bias among participants cannot be ruled out.

Treatment decision may be complex [14], and for each case, the dentist must assess which treatment is the most appropriate. The results from this study comply with this recommendation. The results may indicate that the clinicians evaluate each case individually and base their decisions on what they think is the best for the individual patient. A Cochrane study from 2009 concluded that the rationale for choosing one type of material over another for a particular outcome should be based on clinical efficacy. This is best highlighted by clinical trials, and the absence of such trials to guide clinical decisions in practice is of great concern [31].

## Conclusion

When dentists are treating children with dental caries or DDDs, they must decide the most appropriate treatment in each case. The results of the present study showed a notable disparity between clinician’s treatment choices. In summary, the clinicians based their treatment choice mostly on patient cooperation, prognosis of the tooth and own experience. The results indicate that Norwegian dentists in PDS evaluate each case individually and base their decisions on what they consider is the best for the individual child.

## Abbreviations

AI: Amelogenesis imperfecta
DDD: Dental developmental defect
DF: Dental fluorosis
GIC: Glass ionomer cement
MIH: Molar-incisor hypomineralisation
NSD: Norwegian Center for Research Data
PAMRC: Polyacid-modified glass ionomer cement
PDS: Public Dental Service
RC: Resin composite
RMGIC: Resin-modified glass ionomer cement
SSB: Statistics Norway
SSC: Stainless steel crown
ZOE: Zinc-oxide eugenol
